# *Cordyceps militaris* Extract Protects Human Dermal Fibroblasts against Oxidative Stress-Induced Apoptosis and Premature Senescence

**DOI:** 10.3390/nu6093711

**Published:** 2014-09-16

**Authors:** Jun Myoung Park, Jong Seok Lee, Ki Rim Lee, Suk-Jin Ha, Eock Kee Hong

**Affiliations:** Department of Bioengineering and Technology, Kangwon National University, Chuncheon 200701, Korea; E-Mails: three0313@nate.com (J.M.P.); jongseoklee78@gmail.com (J.S.L.); lkr3410@nate.com (K.R.L.); sjha@kangwon.ac.kr (S.-J.H.)

**Keywords:** *Cordyceps militaris*, apoptosis, fibroblast, oxidative stress, senescence

## Abstract

Oxidative stress induced by reactive oxygen species (ROS) is the major cause of degenerative disorders including aging and disease. In this study, we investigated whether *Cordyceps* *militaris* extract (CME) has* in vitro* protective effects on hydrogen peroxide-induced oxidative stress in human dermal fibroblasts (HDFs). Our results showed that the 2,2-diphenyl-1-picrylhydrazyl (DPPH) radical scavenging activity of CME was increased in a dose-dependent manner. We found that hydrogen peroxide treatment in HDFs increased ROS generation and cell death as compared with the control. However, CME improved the survival of HDFs against hydrogen peroxide-induced oxidative stress via inhibition of intracellular ROS production. CME treatment inhibited hydrogen peroxide-induced apoptotic cell death and apoptotic nuclear condensation in HDFs. In addition, CME prevented hydrogen peroxide-induced SA-β-gal-positive cells suggesting CME could inhibit oxidative stress-induced premature senescence. Therefore, these results suggest that CME might have protective effects against oxidative stress-induced premature senescence via scavenging ROS.

## 1. Introduction

Skin aging can be divided into intrinsic aging (chronologic aging), which is the process of senescence that affects all body organs, and extrinsic aging, which occurs as a consequence of exposure to environmental factors such as sunlight (photo-aging), smoking and dryness [1,2,3]. One of the most important extrinsic aging factors is sunlight, particularly exposure to ultra violet irradiation, which causes skin aging by generating intracellular reactive oxygen species (ROS) in human body [4]. Excessive generation of ROS such as hydroxyl radical (-OH), super oxide anion (O_2_^−^) and hydrogen peroxide (H_2_O_2_) in cells causes cellular homeostasis destruction, oxidative stress and eventually, cell destruction in organs [5]. The “free radical theory of aging” states that progressive defects in cellular function lead to increased production of ROS, which plays a role in the initiation and progression of the aging process [6]. Oxidative stress-induced premature senescence in fibroblasts was recently suggested as an explanation for irreversible growth arrest characterized by senescence-specific cell morphology and enzyme expression [7]. Premature senescence cells, which exposed to hydrogen peroxide, exhibited a state of replicatively senescent cells, such as a large flat morphology and increased senescence-associated β-galactosidase (SA-β-gal) activity [8,9]. Direct exposure of oxidants such as hydrogen peroxide or ultraviolet (UV) radiation can directly induce apoptosis [10,11]. In addition, accumulated ROS have been suggested to play important roles in the photo-aging of human skin* in vivo*, and ROS has been reported to be responsible for various cutaneous inflammatory disorders and skin cancers [12]. Therefore, eliminating intracellular ROS is very important for the prevention and treatment of disease.

*Cordyceps* fungus is a parasitic complex of fungus, which has been used for medicinal purposes of centuries particularly in Korea, China and Asian countries [13].* Cordyceps militaris* (*C. militaris*), an entomophathogenic fungus belonging to the class *Ascomycetes*, has been reported to have beneficial biological activities. Especially, polysaccharides are considered to be one of the major active substances in *C. militaris* and possess various physiological activities; it has been used for multiple medicinal purposes such as hypoglycemic, hypolipidemic, anti-inflammatory, antitumor, anti-metastatic, immunomodulatory, and antioxidant effect [14,15,16,17,18]. In recent years, many cosmetics and skin care products have been supplemented with natural products including mushroom has claimed much attention [19,20]. However, there have been no reports on the preventive effects of *C*.* militaris* on hydrogen peroxide-induced apoptosis and premature senescence in HDFs.

In the present study, we evaluated the preventive effects of a hot-water extract of *C. militaris* on hydrogen peroxide-induced apoptosis and premature senescence in human dermal fibroblasts (HDFs).

## 2. Materials and Methods

### 2.1. Materials

Dulbecco’s Modified Eagle’s Medium (DMEM), penicillin G, streptomycin, and fetal bovine serum (FBS) were obtained from GIBCO (Grand Island, NY, USA). Hydrogen peroxide, 3-(4,5-dimethylthiazol-2-yl)-2,5-diphenyltetrazolium bromide (MTT), 2,2-diphenyl-1-picrylhydrazyl (DPPH) and Hoechst 33342 were purchased from Sigma Biochemicals (St. Louis, MO, USA). 2′,7′-Dichlorodihydrofluorescein diacetate (H_2_DCFDA) and the apoptotic assay kit was purchased from Molecular Probes (Carlsbad, CA, USA). All other chemicals were purchased from Sigma.

### 2.2. Preparation of Cordyceps militaris Extract

The fruiting body of *C. militaris* (20 g) extracted with distilled water (600 mL) at 121 °C for 2 h. The extracts were centrifuged at 5000 RPM for 20 min and filtered through 0.45 μm Whatman filter paper (Whatman Ltd., Maidstone, UK) to remove insoluble matter, and then freeze-dried. The obtained extract of *C. militaris* fruits (CME) was kept at −20 °C until it was used for activity assessment.

### 2.3. Cell Culture

Human dermal fibroblast cells (HDFs) were purchased from American Type Culture Collection (Manassas, VA, USA). HDFs were cultured in DMEM medium supplemented with 10% heat inactivated FBS, 2 mM glutamine, 100 U/mL penicillin, and 100 μg/mL streptomycin. Cells were maintained at 37 °C in a 5% CO_2_ incubator. The cells were cultured to approximately 80% confluence, harvested with 0.25% trypsin-EDTA, then sub-cultured for an additional 48 h in DMEM. For this study, the cells used were early passages from 4 to 10.

### 2.4. DPPH Radical Scavenging Activity

Antioxidative activity of CME was measured according to the DPPH method with slight modifications [21]. CME at different concentrations (10–500 μg/mL) was added to a final 100 μM concentration of DPPH in ethanol. The reaction mixture was shaken for 30 min and then the content of residual DPPH was determined at 520 nm using a microplate reader. The DPPH radical scavenging activity (%) was calculated as follows:

[(optical density of DPPH radical treatment) − (optical density of CME + DPPH radical treatment)]/(optical density of DPPH radical treatment) × 100
(1)

### 2.5. Cell Viability Analysis

To determine the cytotoxic effects of CME, the cell viability of hydrogen peroxide-treated HDFs was determined using the MTT assay, which relies on the ability of viable cells to metabolically reduce the tetrazolium salt MTT to a purple formazan product, which can be quantified colorimetrically. HDFs (2 × 10^4^ cells/mL) were seeded in 12-well plates and incubated for 48 h. Then, CME at different concentrations (10–500 μg/mL) was added to plates. After 24 h, the number of viable cells was determined by MTT assay. To determine the protective effect of CME on hydrogen peroxide-induced cell death, HDFs were seeded in 12-well plates. After 48 h of incubation, the cells were treated with various concentrations of CME for 4 h, followed by addition of 0.8 mM hydrogen peroxide to each well. After 3 h of incubation, 0.5 mg/mL MTT solution was then added, followed by incubation for a further 3 h. Formazan crystals in each well were dissolved in isopropyl alcohol, and the absorbance was determined at 570 nm.

### 2.6. Intracellular ROS Scavenging Activity

Intracellular ROS production was measured with the oxidation-sensitive fluorescent probe; H_2_DCFDA, which is cleaved by intracellular esterases into its non-fluorescent form, DCFH. This form, which is no longer membrane permeable, can be further oxidized by hydrogen peroxide to its fluorescent form, DCF. To investigate the effect of CME on oxidative stress, HDFs (2.5 × 10^4^ cells/well) were seeded in 12-well plates and incubated at 37 °C to allow for cell attachment. After 48 h, the cells were treated with or without CME for 4 h and then 0.8 mM hydrogen peroxide were added to each well of the plate. After 3 h, a 5 μM H_2_DCFDA solution in phosphate-buffered saline (PBS) was added and fluorescence was measured at excitation and emission wavelengths of 485 nm and 535 nm, respectively, by using a microplate spectrofluorometer. For image analysis of intracellular ROS production, HDFs (2.5 × 10^4^ cells/well) were seeded in coverslip loaded 6-well plates and treated in the same manner as above. H_2_DCFDA solution was added to each well of the plate, which was incubated for 2 h at 37 °C. Cells were then fixed with 3.7% paraformaldehyde for 20 min and washed with PBS. After washing with PBS, cells were mounted under glass coverslips with Vectashield (Brunschwig, The Netherlands). Images of the stained cells were collected using a fluorescence microscope (Nikon, Tokyo, Japan).

### 2.7. Measurement of DNA Condensation

The nuclear morphology of the cells was evaluated using the cell-permeable, DNA-specific fluorescent dye Hoechst 33342. Cells with homogeneously stained nuclei were considered to be viable, whereas the presence of chromatin condensation and/or fragmentation was indicative of apoptosis. HDFs were seeded in 12-well plates; then 48 h later, they were treated with various concentrations of CME for 4 h, followed by further incubation for 3 h prior to exposure to 0.8 mM hydrogen peroxide. Then, 5 μg/mL of Hoechst 33342 (stock solution, 10 mg/mL) was added to each well, followed by a 10-min incubation at room temperature. Images of the stained cells were acquired using a Nikon fluorescence microscope in order to examine the degree of apoptotic nuclear condensation and fragmentation.

### 2.8. Apoptosis Analysis

Apoptosis of HDFs was examined using a FITC-labeled annexin V/propidium iodide (PI) apoptosis detection kit according to the manufacturer’s instructions. Briefly, cells were harvested using trypsin-EDTA, washed with PBS, and centrifuged to collect the cell pellets. The cell concentration was adjusted to 1 × 10^6^ cells/mL. The cells were then resuspended in a binding buffer (10 mM HEPES, 140 mM NaCl, 2.5 mM CaCl_2_, pH 7.4) and stained with FITC-labeled annexin V and PI for 15 min at room temperature in the dark. Flow cytometry analysis was performed using a FACS Calibur flow cytometer (Becton Dickinson, Mountain View, CA, USA) within 1 h after supravital staining. FITC-labeled annexin V was analyzed using excitation and emission settings of 488 nm and 535 nm, respectively, and PI was analyzed using excitation and emission wavelengths of 488 nm and 575 nm, respectively. For each flow cytometer run, 10,000 cells were analyzed. The percentages of cells in the defined regions were determined using Cell Quest software (Becton Dickinson). The cells in the early phase of apoptosis were annexin V-positive and PI-negative; however, the cells in the late phase of apoptosis were positive for both annexin V and PI. The percentage of apoptotic cells was calculated as the sum of early and late apoptotic cells divided by the total number of events.

### 2.9. Senescence-Associated β-Galactosidase Staining

Senescence-associated β-galactosidase (SA-β-gal) staining was performed as previously described [22]. Briefly, HDFs were harvested with 0.25% trypsin-EDTA, washed in PBS, fixed with 3.7% paraformaldehyde for 15 min at room temperature, washed with PBS, and then incubated in freshly prepared SA-β-gal staining solution (1 mg/mL 5-bromo-4-chloro-3-indolyl β-d-galactopyranoside, 5 mM potassium ferrocyanide, 2 mM magnesium chloride, pH 6.0) at 37 °C overnight. At the end of the incubation, the cells were washed with PBS. Senescent cells were identified as blue-stained cells by standard light microscopy at 100-fold magnification, and a total of 200 cells were counted in five random fields on a slide to determine the percentage of positively stained cells.

### 2.10. Statistical Analysis

Data are expressed as means ± the standard error of the mean (SEM), and the results were derived from at least three independent experiments performed in triplicate. The data were analyzed using Student’s *t*-test to evaluate the significance of differences; *p* < 0.05 was regarded as statistically significant.

## 3. Results

### 3.1. DPPH Radical Scavenging Activity of CME

**Figure 1:**
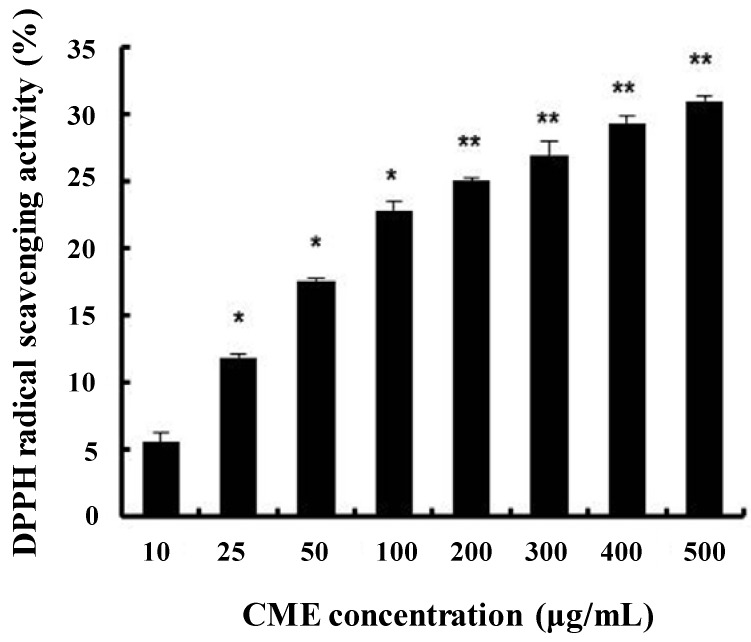
DPPH radical scavenging activity of *Cordyceps militaris* extract (CME). The amount of DPPH radicals was determined spectrophotometrically at 520 nm. Data represent the mean ± SE of three independent experiments. Significance of the differences were compared with the non-treated group at * *p* < 0.05.

To evaluate the antioxidative activity of CME, the method of DPPH-radical scavenging activity was performed. CME significantly increased the DPPH radical scavenging activity in a dose-dependent manner (Figure 1). Several researchers have investigated the antioxidative activity of flavonoid compounds and have attempted to define the structural characteristics of flavonoids that contribute to their antioxidant activity [23,24]. Some reports have suggested that C. militaris exhibits potent free radical scavenging effects [25]. From these results, we suggest that CME has antioxidant activity.

### 3.2. Cytoprotective Effect of CME against Hydrogen Peroxide-Induced Oxidative Stress in HDFs

**Figure 2:**
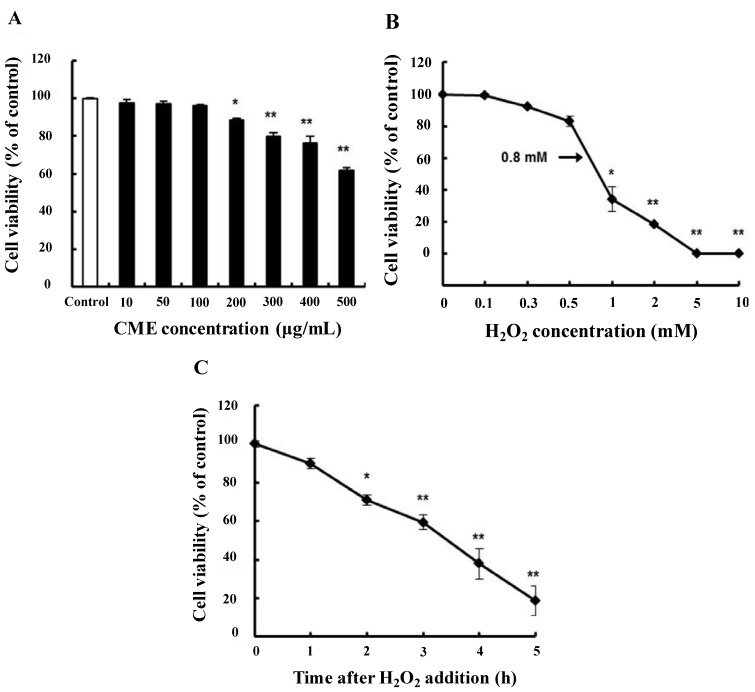
Viability of human dermal fibroblasts (HDFs) treated with CME or H_2_O_2_. (**A**) Cell viability of HDFs treated with various concentration of CME; (**B**) cell viability of HDFs treated with various concentrations of H_2_O_2_ for 3 h; (**C**) cell viability of HDFs treated with different exposure time with 0.8 mM H_2_O_2_. Cell viability was determined using the MTT assay. Data represent the mean ± SE of three independent experiments. Significance of the differences were compared with the control at * *p* < 0.05 and ** *p* < 0.01.

Dose-response experiments were carried out in order to determine the maximum non-toxic concentration of CME to be used. MTT assays showed that there was no significant change in cell viability after treatment with CME at concentrations ranging from 10 to 200 μg/mL. At a concentration of CME 200 μg/mL, cell viability was approximately 89% (Figure 2A). Many studies have demonstrated the cytotoxic effects of oxidizing agents such as hydrogen peroxide on human fibroblasts [26,27].

**Figure 3:**
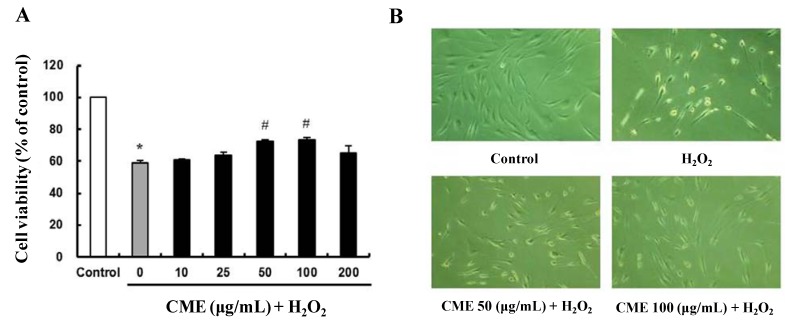
Protective effect of CME on H_2_O_2_-treated HDFs. (**A**) Cells were exposed to 10–200 μg/mL of CME for 4 h, then 0.8 mM H_2_O_2_ for 3 h. Cell viability was determined using the MTT assay. Data represent the mean ± SE of three independent experiments. The significance of differences as compared with the control group (* *p* < 0.05) and the H_2_O_2_ alone group (**^#^** *p* < 0.05) were assessed using Student’s *t*-test. (**B**) Morphological changes of HDFs after various treatments were observed by optical microscopy. The conditions were control (no treatment), 0.8 mM H_2_O_2_ for 3 h, 0.8 mM H_2_O_2_ for 3 h with 50 μg/mL of CME, and 0.8 mM H_2_O_2_ for 3 h with 100 μg/mL of CME. Magnification: 400×.

Hydrogen peroxide was used to induce oxidative stress in HDFs, and it reduced cell viability in a concentration- and time-dependent manner. After incubation with 0.8 mM hydrogen peroxide for 3 h, cell viability was reduced by approximately 59% compared to the control (Figure 2B,C). In order to assess the protective effect of CME on oxidative stress-damaged HDFs, the MTT assays were performed. In comparison to the control, 0.8 mM hydrogen peroxide caused a significant reduction in cell viability by 59% (Figure 3A). However, pretreatment of CME (50 or 100 μg/mL) for 4 h in HDFs resulted in the prevention of cell death induced by hydrogen peroxide. To confirm the protective effect of CME on cell survival in hydrogen peroxide-treated cells, we performed a morphology study. Cell morphology changes with hydrogen peroxide treatment in HDFs were observed (Figure 3B). Compared to control cells, treatment with hydrogen peroxide for 3 h resulted in abnormal morphology such as cell shrinkage. However, pretreatment with CME at different concentrations (50 or 100 μg/mL) reduced this abnormal morphology. Thus, we suggest that CME is capable of preventing oxidative stress-induced cell death in HDFs.

### 3.3. CME Inhibited Hydrogen Peroxide-Induced ROS Generation in HDFs

**Figure 4:**
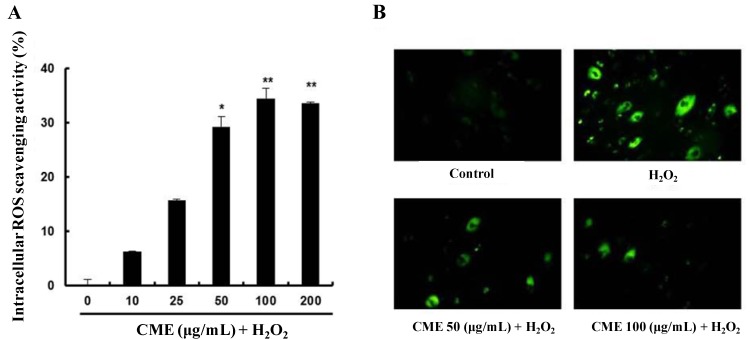
Intracellular reactive oxygen species (ROS) scavenging activity of CME in HDFs. (**A**) CME was added at 10–200 μg/mL for 4 h followed by addition of 0.8 mM H_2_O_2_ for 3 h. Intracellular ROS scavenging activity was measured using the H_2_DCF-DA method. Data represent the mean ± SE of three independent experiments. The significance of differences between H_2_O_2_ alone group and CME-treated groups was assessed using Student’s *t*-test. ** *p* < 0.01. (**B**) Representative fluorescence images illustrating the increase in green fluorescence intensity of DCF produced by ROS in H_2_O_2_-treated cells as compared to control and cells treated with CME. The conditions were control (no treatment), 0.8 mM H_2_O_2_ for 3 h, 0.8 mM H_2_O_2_ for 3 h with 50 μg/mL of CME, and 0.8 mM H_2_O_2_ for 3 h with 100 μg/mL of CME. Magnification: 400×.

To investigate intracellular ROS scavenging activity of CME, the ROS sensitive fluorescent probe, H_2_DCFDA was used. As shown in Figure 4A, pretreatment with CME for 4 h significantly increased intracellular ROS scavenging activity. In the fluorescent microscope images, the fluorescence intensity was enhanced in H_2_O_2_-treated HDFs. However, pretreatment with CME for 4 h highly decreased the fluorescence intensity (Figure 4B). These results suggest that CME might prevent hydrogen peroxide-induced oxidative stress through ROS scavenging activity.

### 3.4. Effect of CME on Hydrogen Peroxide-Induced Apoptotic Cell Death in HDFs

**Figure 5:**
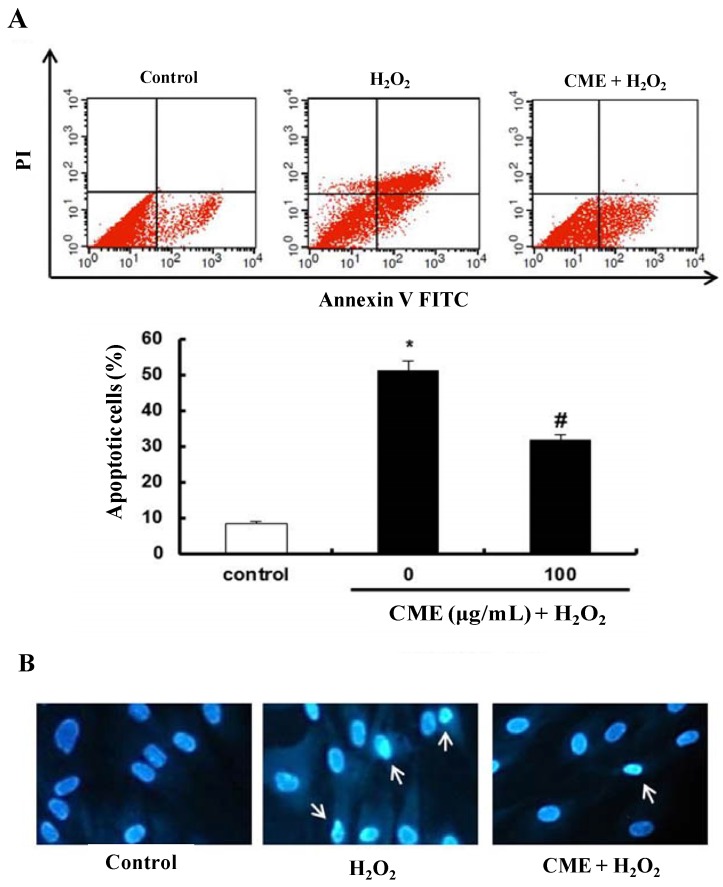
Protective effect of CME on hydrogen peroxide-induced apoptosis in HDFs. (**A**) Apoptotic cells were detected by annexin V and PI double staining and analyzed by flow cytometry. The conditions were control (no treatment), 0.8 mM H_2_O_2_ for 3 h, and 0.8 mM H_2_O_2_ for 3 h with CME (100 μg/mL). The bottom graphs represent the percentage of apoptotic-cell death as indicated by the total number of cells in the upper right and lower right quadrants. Data represent the mean ± SE of three independent experiments. The significance of differences as compared with the control group (* *p* < 0.05) and the H_2_O_2_ alone group (^#^ *p* < 0.05) were assessed using Student’s *t*-test. (**B**) DNA condensation of HDFs was detected by Hoechst 33342 staining. The conditions were control (no treatment), 0.8 mM H_2_O_2_ for 3 h, and 0.8 mM H_2_O_2_ for 3 h with CME (100 μg/mL). Apoptotic cells with blue nucleus showing condensation of chromatin as dense blue areas (white arrows). Magnification: 400×.

To investigate whether CME have the cytoprotective effect on hydrogen peroxide-induced cell damage, a double-staining method using FITC-labeled annexin V and PI was used to detect apoptotic cells by flow cytometry. A significant increase in the apoptotic rate (from 8.5% to 51.3%) was observed after treatment with hydrogen peroxide for 3 h in HDFs. However, pretreatment with 100 μg/mL CME significantly inhibited hydrogen peroxide-induced apoptosis in HDFs (Figure 5A). CME-only treated group was not affected (data not shown). To detect the extensive DNA condensation characteristic of apoptotic cell death, Hoechst 33342 staining was conducted. As shown in Figure 5B, hydrogen peroxide treatment caused a significant increase in fluorescence intensity and deviation of stained DNA, indicating DNA condensation. Pretreatment with CME decreased hydrogen peroxide-induced DNA condensation. Therefore, these results suggest that CME might have cytoprotective effects on hydrogen peroxide-induced apoptotic cell death.

### 3.5. Effect of CME on Hydrogen Peroxide-Induced Premature Senescence in HDFs

**Figure 6:**
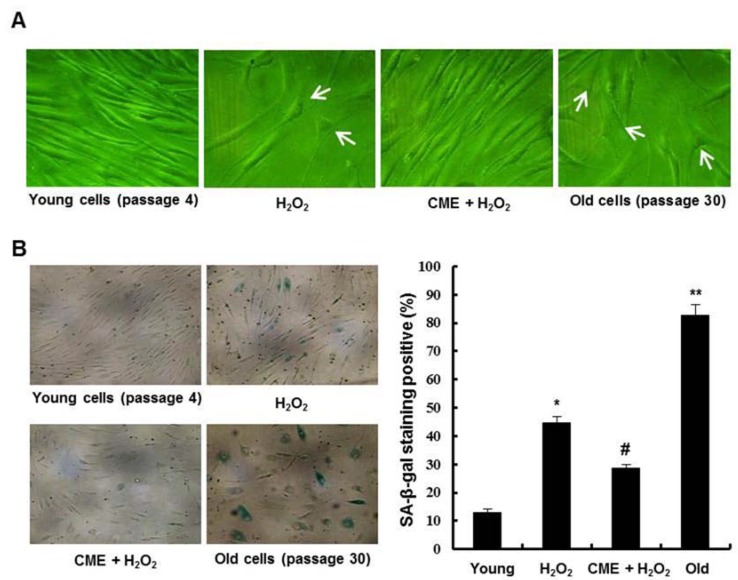
Inhibitory effect of CME on oxidative stress-induced premature senescence in HDFs. The cells were treated with 0.8 mM H_2_O_2_ for 3 h in the presence or absence of 100 μg/mL of CME and then recovered for 7 days with fresh medium. (**A**) Morphological observation of cells was undertaken via optical microscopy. Magnification: 200×. (**B**) Representative micrographs of young cells (passage 4), 0.8 mM H_2_O_2_-induced premature senescent cells, CME-treated cells, and old cells (passage 30) after senescence-associated β-galactosidase (SA-β-gal) staining. Magnification: 100×. The percentage of SA-β-gal positive cells was quantified (right graph). Data represents the mean ± SE of three independent experiments. The significance of differences as compared with the young cells group (* *p* < 0.05, ** *p* < 0.01) and the H_2_O_2_ alone group (^#^ *p* < 0.05) were assessed using Student’s *t*-test.

To determine the effects of CME on hydrogen peroxide-induced premature senescence in HDFs, we investigated the phenotypic observations associated with senescence. Cells treated with hydrogen peroxide exhibited characteristics of a senescent phenotype such as enlarged and flatted cell morphology (white arrows). However, cells treated with CME prior to hydrogen peroxide exposure exhibit a reduction of these phenomena (Figure 6A). CME-only treated group was not affected (data not shown). We observed that hydrogen peroxide treatment in HDFs accumulated the SA-β-gal activity in the cells. Pretreatment with CME prior to exposure to hydrogen peroxide inhibited SA-β-gal positive cells (Figure 6B). Thus, these results suggest that CME inhibited oxidative stress-induced premature senescence in HDFs.

## 4. Discussion

Increased exposure to UV irradiation represents a major environmental threat to the skin, increasing its risk of photo-oxidative damage by UV-induced ROS [28,29]. ROS activate cytoplasmic signal transduction pathways in fibroblasts that are related to differentiation, senescence, and connective tissue degradation [30]. Increased ROS results in several pathological states including photo-aging and photo-carcinogenesis of the skin. Therefore, the ability to scavenge the ROS in cells is important for cellular senescence [31]. The ability to scavenge the ROS in cells is important for cellular senescence. Recent studies have shown that natural compounds protect from oxidative stress-induced damage in fibroblasts [32,33]. There is a significant amount of experimental evidence suggesting *C. militaris* has antioxidant activity [34]. However, the potential roles of *C. militaris* in conferring protection against oxidative stress-induced premature senescence have not been well demonstrated.

Some evidence provided that human dermal fibroblasts in culture have been used as* in vitro* models for the study of aging. Under standard culture conditions or after the action of known stressors as oxidants, cells exhibit several features, of which cell cycle arrest is the loss-of-function hallmark [35]. As a result of exposure to hydrogen peroxide, cells exposed to hydrogen peroxide exhibited distinct morphological features indicative of apoptosis, including membrane blebbing, increased annexin V/PI positive staining and DNA condensation [36]. In addition, various studies have demonstrated that elevated levels of intracellular ROS have been implicated in Bcl-2 family protein such as Bax and Bak [37,38]. The generation of ROS, such as superoxide anion and hydrogen peroxide, initiate the mitochondria-mediated apoptotic pathway by altering expression of Bcl-2 family protein, thereby increasing changing the mitochondrial transmembrane potential, affecting mitochondrial membrane integrity and releasing of cytochrome c. In the cytosol, cytochrome c forms an apoptosome that is composed of Apaf-1 and pro-caspase-9, resulting in the activation of caspase-9 [39,40]. Caspase-9 activates the effector pro-caspases, including pro-caspase-3, to carry out the process of apoptosis [41]. In the present study, we detected an increase in apoptotic cells and DNA condensation after hydrogen peroxide treatment in HDFs. However, cells treated with CME showed less DNA condensation and a reduction in apoptosis (Figure 5). These results indicated that CME functions as an anti-apoptosis, by reducing intracellular ROS production. Therefore, we suggest that CME prevent hydrogen peroxide-induced apoptotic cell death by ROS scavenging activity.

Oxidative stress leads to a state of stress-induced premature senescence which displays features of replicative senescence such as lack of cell proliferation, increased cell volume, distinct flat morphology, and elevated expression of cell cycle inhibitor proteins [42,43]. Moreover, the presence of senescence-associated β-galactosidase (SA-β-gal) activity has been a widely used biomarker of cellular senescence [44]. In this study, cells treated with hydrogen peroxide exhibited characteristics of a senescent phenotype such as increased cell volume, flattened cell morphology, and higher SA-β-gal activity (Figure 6). However, cells pretreated with CME prior to hydrogen peroxide exposure exhibit a reduction of these phenomena. Thus, these results suggest that CME inhibited oxidative stress-induced premature senescence via its ability to scavenge ROS.

## 5. Conclusions

In conclusion, our results suggest that CME possesses ROS scavenging activity, inhibits hydrogen peroxide-induced apoptosis, and suppresses premature senescence in human dermal fibroblasts. Hence, we provided evidence that CME had potent anti-aging activity* in vitro*. Further clarification of the correlation between structure and the antioxidative activities of *C. militaris*, mechanistic studies of the protective signaling pathway will be the subject of subsequent investigation.
